# Protein supplementation and resistance training-induced strength gains in young adults: a meta-analysis

**DOI:** 10.3389/fnut.2026.1946052

**Published:** 2026-10-13

**Authors:** Chenkun Zhang, Yahai Wang, Wenying Huang

**Affiliations:** 1 Department of Physical Education, Science and Technology College of Nanchang University, Jiujiang, Jiangxi, China; 2 College of Physical Education, Jiangxi Normal University, Nanchang, China; 3 College of Arts and Physical Education, Nanchang Normal College of Applied Technology, Nanchang, China

**Keywords:** exercise performance, meta-analysis, muscle strength, protein intake, resistance training

## Abstract

**Background:**

This study aimed to determine whether protein supplementation confers additional benefits for muscle strength gains beyond those induced by resistance training (RT) alone in healthy young adults.

**Methods:**

A systematic literature search was performed in PubMed, Web of Science, Cochrane Library, and SPORTDiscus through April 1, 2026. Twenty-three randomized controlled trials involving 611 healthy young adults were included. Pooled estimates were calculated as weighted mean differences (WMD) with 95% confidence intervals (CIs) using a random-effects model. Subgroup analysis, meta-regression, and leave-one-out and leave-one-category-out sensitivity analysis were performed to explore heterogeneity.

**Results:**

Compared with RT alone, combined RT and protein supplementation produced significant improvements in both bench press (WMD: 2.63 kg, 95% CI: 0.77–4.49, *p* = 0.006) and squat strength (WMD: 7.40 kg, 95% CI: 2.15–12.66, *p* = 0.006). Subgroup analysis revealed that the ergogenic effect on squat strength was predominantly observed when the intervention lasted ≤12 weeks, or when protein was ingested both around exercise (pre- and/or post-exercise) and before sleep. Meta-regression identified intervention duration as a significant moderator (*p* = 0.037) and protein timing as a borderline moderator (*p* = 0.073) of squat strength heterogeneity. Sensitivity analysis showed that the pooled effects remained significant and consistent in direction.

**Conclusion:**

Protein supplementation combined with resistance training enhances muscle strength in young healthy adults, but its incremental benefit appears conditional on intervention duration, nutrient timing, training background, and baseline protein status rather than universally applicable. Future well-powered trials with standardized protein dose and source reporting, repeated measures of habitual protein intake, and stratified designs are needed to clarify these interactions and support more personalized, mechanism-informed exercise and nutrition guidance.

**Systematic review registration:**

https://www.crd.york.ac.uk/PROSPERO/# myprospero, PROSPERO, identifier: CRD42024534481.

## Introduction

Age-related decline in muscle strength is a well-documented phenomenon and a critical marker of physical health in adults (1–3). Sedentary individuals as well as those who engage in prolonged periods of inactivity or lack of physical exercise might expedite the deterioration of muscle strength (4). Resistance training (RT) has been established as an effective intervention to mitigate this decline by stimulating muscle protein synthesis and enhancing neuromuscular adaptation (5–7). Concurrently, protein supplementation is widely advocated to augment the anabolic response to RT, particularly by accelerating muscle protein synthesis rates post-exercise (8–10). However, the potential additive effects of protein supplementation alongside RT remain a topic of debate, with conflicting findings across randomized controlled trials (RCTs) and meta-analysis (11–17).

While some studies suggest that combining protein supplementation with RT leads to greater strength gains compared to RT alone (11, 15–17), others report no significant additional benefits (12–14). These discrepancies may arise from variations in study design, including differences in protein dosage, timing (e.g., pre- vs. post-exercise), type (e.g., whey, casein, plant-based), and intervention duration. Furthermore, previous meta-analysis have often included mixed populations (e.g., older adults, athletes) or lacked standardized strength assessments (e.g., using heterogeneous measures like 1RM leg press vs. squat), limiting their applicability to young, healthy individuals.

To address these gaps, this meta-analysis aimed to quantitatively evaluate the efficacy of combining RT with protein supplementation versus RT alone in improving muscle strength—measured via bench press (upper-body) and squat (lower-body) performance—in young healthy adults. We further stratified results by key moderators (e.g., supplementation timing, intervention duration) to identify optimal practices for maximizing strength adaptations. By synthesizing data from 23 RCTs, this study provides evidence-based insights into the role of protein supplementation in RT regimens in this population, bridging the gap between research and practical application in exercise and nutrition science.

## Methods

This meta-analysis was conducted and documented in accordance with the principles outlined in the Preferred Reporting Items for Systematic Reviews and Meta-Analysis 2020 (PRISMA 2020) (18). The study protocol was formally documented in the PROSPERO, which is the International Prospective Register of Systematic Reviews. (CRD 42024534481).

### Search strategy

We performed electronic searches on PubMed, Cochrane Library, Web of Science, and SPORTDiscus databases to find papers that were relevant to our study. We used the search terms “randomised controlled trials,” “protein supplements,” “resistance training,” and “muscle strength.” In addition, we conducted a manual search of the reference lists of relevant papers and reviews to find any supplementary investigations. The search was limited to research published in English from the beginning until April 1st, 2026. The comprehensive search approach was displayed in Supplementary Table S1.

### Study selection

In order to be selected, research needed to meet the following criteria: (1) The study involved randomized controlled trials with either a parallel or crossover design, conducted on health adults (<45 years old). (2) The intervention being studied was protein supplementation in combination with RT, and the comparison groups were either RT alone or RT with a placebo. (3) The outcome measures of interest were the net changes in muscle strength, along with their corresponding standard deviations (SDs), or enough information was provided to calculate them. Studies were excluded if they met any of the following criteria: (1) The experiments were not randomized controlled trials. (2) The individuals in the treatment group or control group did not undergo resistance training. (3) There is a lack of data on the changes in muscle strength or the availability of data to calculate it. (4) Protein was included in the meals of the control group. When numerous studies contained duplicate data from the same trial, we only considered the study that provided the most comprehensive information.

### Data extraction and quality assessment

The research selection and data extraction were conducted autonomously by two investigators using a standardized form. Discrepancies were handled through deliberation with the third reviewer in order to achieve an agreement. The review included eligible studies and collected the following information: first author’s name, publication year, participants’ health status, sample size, participants’ age and sex, duration of the study, comparator used, information on RT (frequency and intensity), details of protein intervention (type, daily dose, and timing of intake), and outcomes measured (Bench press and Squat). We extracted just the data from the longest follow-up for a study that reported multiple data at different time periods during the whole investigation. If a study featured two separate intervention strata, such as different dosages or supplements, it was considered as two separate reports. The PEDro scale was used to assess the methodological quality and risk of bias of each study included in the analysis (19).

### Statistical analysis

The random-effect model was recommended for calculating the overall effect magnitude. The pooled effect sizes were calculated as the weighted mean difference (WMD) along with 95% confidence intervals (CI) for the net changes in muscle strength (kg). When a study contributed multiple eligible intervention arms (e.g., different protein types, doses, or timing strategies), each arm was treated as a separate intervention group. If these arms shared the same control group, the shared control group was used for comparison with each intervention arm, and control participants were counted only once when calculating the pooled sample size. To account for the potential dependency between comparisons, we performed a sensitivity analysis in which the shared control group sample size was divided equally across the relevant comparisons for multi-arm studies, while control means and standard deviations remained unchanged. The net changes were determined by subtracting the control group values from the intervention group values, and then calculating the difference between the final values and the baseline values of the mean values. If there are no reported standard deviations (SDs) for changes from baseline in each group, they will be estimated using the following formula: The standard deviation (SD) is calculated as the square root of the sum of the squares of the pre-treatment SD (SDpre-treatment) and the post-treatment SD (SDpost-treatment), minus twice the product of the correlation coefficient (R) and the product of the pre-treatment and post-treatment SD (SDpre-treatment and SDpost-treatment, respectively) (SD = square root [(SDpre-treatment)^2^ + (SDpost-treatment)^2^ − (2R × SDpretreatment × SDpost-treatment)]). In this calculation, a correlation coefficient of 0.8 is assumed. When standard errors (SEs) were supplied instead of SD_S_, we transformed the SEs to SDs for additional analysis using the formula: SD = SE × sqrt (n). In studies where data were presented as medians and interquartile ranges (IQRs), the means and related standard deviations (SDs) were calculated using the approach given by Wan et al. (20).

The heterogeneity among studies was assessed using statistical methods such as I^2^ and Cochrane’s Q test. Heterogeneity was considered present when the I^2^ value exceeded 50% and the P value for the Q test was less than 0.1. To investigate the occurrence of heterogeneity, we utilized subgroup analysis and meta-regression to uncover plausible factors contributing to the heterogeneity. To account for publication bias, a funnel plot and Egger’s regression test were both utilized (21). A sensitivity analysis was conducted using leave-one-out analysis to evaluate the reliability of the main outcome. The analysis were conducted using STATA version 16.0 (Stata Corp, College Station, TX, USA). A *p*-value less than 0.05 was considered statistically significant, unless stated otherwise.

### Certainty assessment

The assessment of the overall quality of outcome evidence is determined using the Recommendation, Evaluation, Development, and Evaluation Grading (GRADE) method (22). GRADE guidelines classify randomized controlled trials (RCTs) as high quality by default, but the degree of evidence can be decreased by variables such as unexplained heterogeneity or increased by characteristics such as a significant size of effect (22).

## Results

### Literature search

The comprehensive procedure of conducting a literature search and selecting relevant studies is illustrated in flowchart Figure 1. A total of 11,596 publications (4,538 from PubMed, 2,658 from Web of Science, 3,848 from Cochrane Library, and 552 from SPORTDiscus) were initially found in the primary database search. Out of these, 3,715 studies were excluded due to duplication. After reviewing the titles and abstracts of the remaining research, 7,584 studies were deemed irrelevant and excluded, while 3 studies were unable to be accessed. A total of 96 publications were omitted from the study because they did not comply with the research design requirements. Additionally, 106 papers were excluded because they had inadequate intervention methods, and 69 articles were excluded because they used improper outcome indicators. The final meta-analysis will contain the remaining 23 studies (23–45).

**Figure 1:**
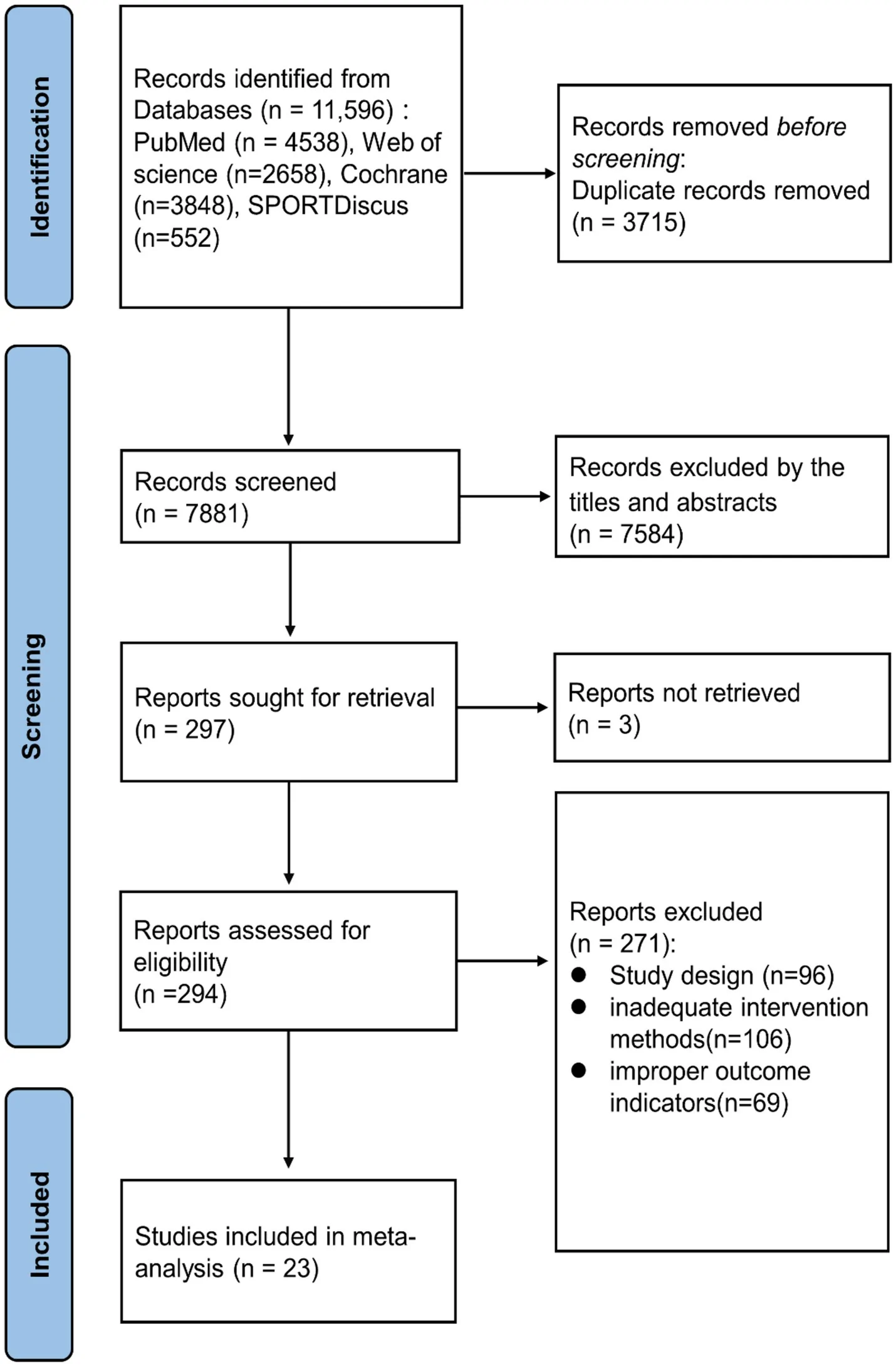
Flow chart of study selection.

### Study characteristics

Table 1 provided a concise overview of the characteristics of the 23 studies that were considered. The meta-analysis comprised 611 healthy adult individuals, with a mean age of 25.32 years. The maximum sample size was 63 participants, and the minimum sample size was 12 individuals. The intervention time ranged from 6 weeks to 36 weeks. Eleven studies did not report the outcome of squat. The frequency of RT in each trial was consistently maintained at a minimum of 3 days per week. Whey protein was the most often utilized form of protein intake, whereas placebo was the most regularly employed substance in the control group.

**Table 1:** Characteristics of included studies.

Study	Subjects	N (I/C)	Age	Duration (wk)	Comparator	RT intervention	Protein type	Protein supplementation (g/d; g/kg/d)	Outcomes (1RM)	Protein intake time on EX-days	Quality^a^
Candow, 2006 (23)	Untrained healthy subjects	27 (9, 9/9)	23.17	12	P (maltodextrin)	3 × per 4 days, 60–90% 1RM	Whey	/; 1.2	Bench press, Squat	AE, BE, BS	Excellent
Candow, 2008 (24)	Healthy men	23 (10/13)	42.30	10	C	3 × per wk., 70% 1RM	/	/; 0.3	Bench press	AE, BE, BS	Excellent
Cribb, 2007 (25)	Recreational male body-builders	12 (5/7)	24.00	11	P (CHO)	3 × per wk., 70–95% 1RM	Whey	103; 1.5	Bench press, Squat	midmorning, AE, BS	Excellent
DeNysschen, 2009 (26)	Healthy men	28 (9; 10/9)	38.00	12	P (CHO)	3 × per wk., /	soy, whey	25.8; 1.2	Bench press, Squat	AE	Excellent
Farnfield, 2012 (27)	Healthy men	16 (8/8)	20.45	12	P (aspartame sweetener)	3 × per wk., 50–80% 1RM	Whey	26.6, /	Bench press	AE	Good
Hoffman, 2007 (28)	Healthy male strength and power athletes	21 (11/10)	20.63	12	P (maltodextrin)	4 × per wk., /	mixture	84; /	Bench press, Squat	morning, AE	Good
Hoffman, 2009 (29)	Healthy male strength and power athletes	33 (13; 13/7)	19.6	10	C	4 × per wk., /	mixture	42; /	Bench press, Squat	morning, evening	Good
Josse, 2010 (30)	Healthy women	20 (10/10)	22.80	12	P (maltodextrin)	5 × per wk., 80–90% 1RM	fat-free milk	18; /	Bench press	AE, AE+1 h	Excellent
Kerksick, 2006 (31)	Healthy men	21 (10/11)	31.00	10	P (CHO)	4 × per wk., /	Whey, casein	40; /	Bench press	AE	Excellent
Kirmse, 2019 (32)	Young men	57 (29/28)	24.00	12	P	3 × per wk., 50–70% 1RM	collagen	15; /	Bench press, Squat	AE	Excellent
Lockwood, 2016 (33)	Healthy resistance-trained men	28 (13/15)	21.40	8	P (dextrose)	4 × per wk., 60–80% 1RM	Whey	30; /	Bench press	AE, BE	Excellent
Naclerio, 2017 (34)	Regularly physically active men	24 (8; 8/8)	27.00	8	P (CHO)	3 × per wk., /	beef, whey	20; /	Bench press, Squat	AE	Excellent
Negro, 2014 (35)	Healthy adults	26 (12/14)	23.81	8	C	3 × per wk., 75% 1RM	Beef	20; /	Bench press	AE	Good
Obradovic, 2020 (36)	Healthy trained men	30 (15/15)	27.00	8	C	4 × per wk., 80–95% 1RM	Whey	/; 1.0	Bench press, Squat	AE, BE	Good
Pourabbas, 2021 (37)	Trained young males	30 (15/15)	27.00	6	P (maltodextrin)	4 × per wk., 60–90% 1RM	Whey, soy	10; /	Bench press	AE, BS	Excellent
Rankin, 2004 (38)	Healthy untrained men	19 (10/9)	20.74	10	P (CHO)	3 × per wk., /	Milk	/; 0.21	Bench press	AE	Excellent
Sharp, 2017 (39)	Men and women	41 (10; 10; 11/10)	20.76	8	P (maltodextrin)	3 × per wk., /	Milk	46; /	Bench press	AE	Excellent
Taylor, 2016 (40)	Female basketball players	14 (8/6)	20.43	8	P (maltodextrin)	2 × per wk., /	mixture	/; 2	Bench press	AE, BE	Excellent
Thomas, 2011 (41)	Healthy women	29 (15/14)	36.80	16	P (sucrose)	3 × per wk., 60–100% 1RM	Yogurt	/; 5.0	Bench press, Squat	AE, BE	Excellent
Vangsoe, 2018 (42)	Healthy young men	18 (9/9)	24.20	8	P (CHO)	4 × per wk., 70% 1RM	insect protein	/; 0.8	Bench press	AE, BS	Good
Volek, 2013 (43)	Non-resistance-trained men and women	63 (19; 22/22)	23.04	36	P (maltodextrin)	/	Whey	21.6; /	Bench press, Squat	AE	Excellent
Willoughby, 2006 (44)	Untrained men	19 (10/9)	19.00	10	P (dextrose)	4 × per wk., 85–90% 1RM	Whey, casein	40; /	Bench press	AE, BE	Excellent
Zbinden-Foncea, 2023 (45)	Untrained young males	12 (6/6)	22.4	8	P (Placebo Juice)	3 × per wk., 75% 1RM	Whey	30; /	Bench press	AE	Excellent

^a^Better methodological quality is indicated by a higher PEDro score (9–10: excellent; 6–8: good; 4–5: fair; <4: poor). AE, after exercise; BE, before exercise; BS, before sleep; C, control; EX, exercise; P, placebo.

### Effect of protein supplementation on muscle strength

A total of 23 studies with 32 intervention groups examined the synergistic impact of protein supplementation and RT on bench press performance in healthy adults. Taking into account the range of effects, we employed the random-effect model to obtain the combined estimate. When comparing the group that only did RT with the group that did protein supplements + RT, there was a noticeable improvement in bench press (WMD: 2.63 kg, 95% CI: 0.77–4.49, *p* = 0.006) (Figure 2). The analysis of I^2^ and Cochrane’s Q test revealed a significant level of heterogeneity (I^2^ = 92.8%; *p* < 0.001) (Table 2). And 12 studies, which included 15 interventions, reported the collective impact of protein supplementation and RT on squat performance in healthy adults. When comparing the group that only did RT with the group that did protein supplementation + RT, there was a noticeable improvement in squat (WMD: 7.40 kg, 95% CI: 2.15–12.66, *p* = 0.006) (Figure 3). The results of I^2^ and Cochrane’s Q test demonstrated there was substantial heterogeneity (I^2^ = 93.9%; *p* < 0.001) (Table 2).

**Figure 2:**
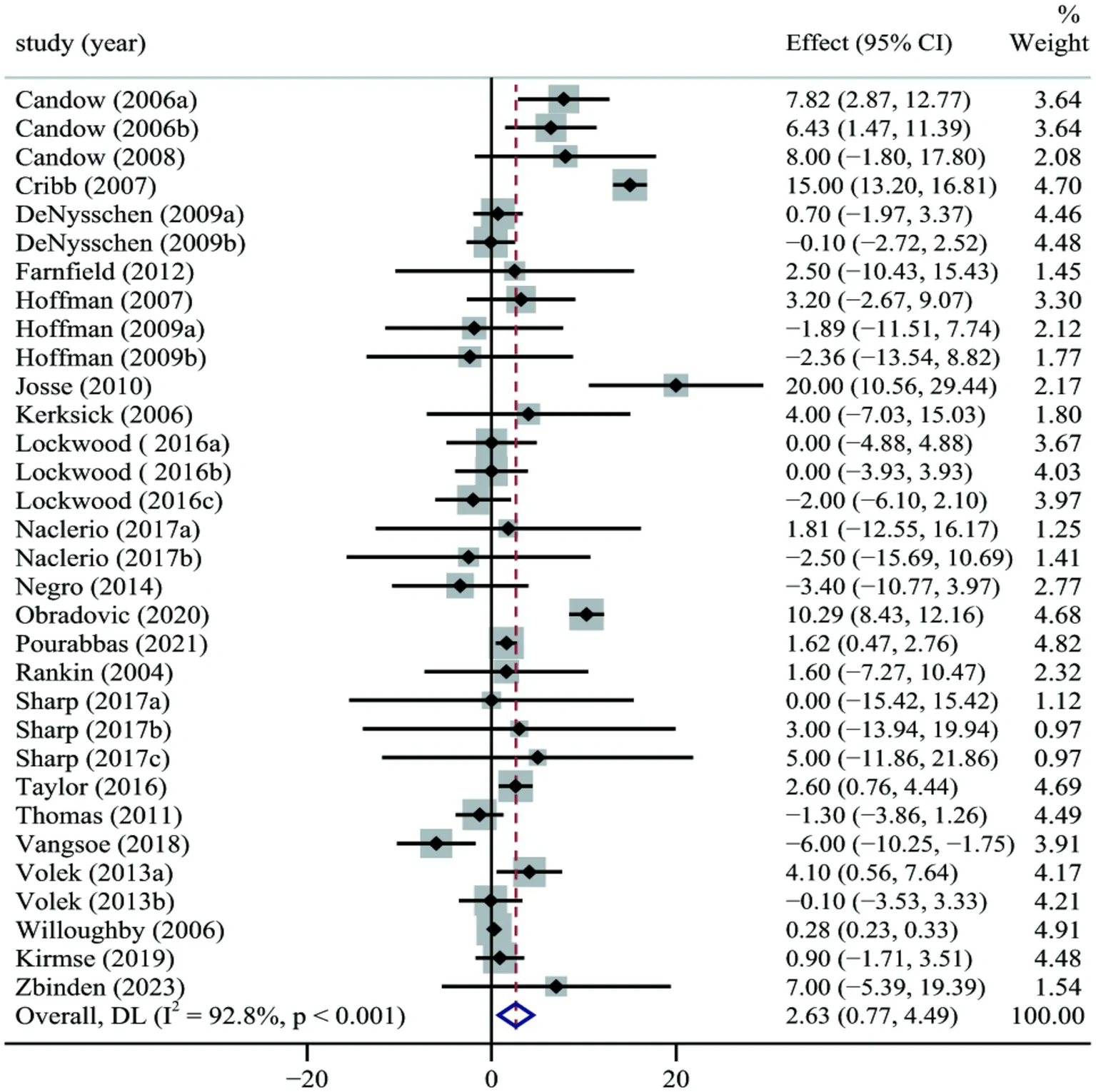
The forest plot of the effect of resistance training combined with protein supplementation on bench press strength in young healthy adults.

**Table 2:** Results of subgroup and meta-regression analysis stratified by study characteristics.

Outcomes (1RM)	Interventions	Mean difference (95% CI)	*p*^1^ (effect)	Heterogeneity		*p*^3^ (meta-regression)	*p* ^4^	
				I^2^ (%)	*p* ^2^		Begg’s value	Egger’s value
Bench press								
Overall	32	**2.63 (0.77, 4.49)**	**0.006**	**92.8**	**<0.001**	**–**	0.697	0.055
Age						0.767	–	–
Gender						0.619		
Men	21	2.15 (−0.31, 4.60)	0.087	94.8	**<0.001**	–	–	–
Women	3	4.65 (−1.33, 10.64)	0.128	90.3	**<0.001**	–	–	–
Mix	8	**3.22 (0.34, 6.11)**	**0.028**	**42.0**	**0.099**	**–**	**–**	**–**
Duration						0.846		
≤12 weeks	29	**2.89 (0.81, 4.96)**	**0.006**	**93.4**	**<0.001**	**–**	**–**	**–**
>12 weeks	3	0.74 (−2.41, 3.89)	0.646	66.5	0.050	–	–	–
Protein type						0.295		
Whey	13	**3.48 (0.66, 6.31)**	**0.015**	**96.4**	**<0.001**	**–**	**–**	**–**
Others	18	1.03 (−1.17, 3.24)	0.262	**58.5**	**0.002**	**–**	**–**	**–**
Training status								
Untrained	19	**1.92 (0.46, 3.38)**	**0.010**	**58.6**	**0.001**	0.673		
Recreational	4	2.38 (−12.02, 16.78)	0.746	**96.5**	**<0.001**	–		
Well-trained	9	1.94 (−1.16, 5.03)	0.220	**89.4**	**<0.001**	–		
Protein time						0.606		
AE	16	1.66 (−0.17, 3.48)	0.076	33.9	0.091	–	–	–
AE + BE	8	1.60 (−1.66, 4.86)	0.336	**94.1**	**<0.001**	–	–	–
AE + BE+BS	3	**7.23 (3.93, 10.52)**	**<0.001**	0.00	0.915	–	–	–
Others	5	2.47 (−5.23, 10.17)	0.529	**97.9**	**<0.001**	–	–	–
Squat								
Overall	15	**7.40 (2.15, 12.66)**	**0.006**	**93.9**	**<0.001**	–	0.882	0.125
Age						0.819	–	–
Gender						0.122	–	–
Men	10	**10.64 (4.47, 16.81)**	**0.001**	**93.8**	**<0.001**	–		
Women	1	−1.60 (−5.88, 2.68)	0.463	–	–	–		
Mix	4	2.78 (−5.88, 11.44)	0.530	**84.8**	**<0.001**	–		
Duration						**0.037**		
≤12 weeks	12	**10.64 (5.42, 15.86)**	**0.001**	**92.6**	**<0.001**	–	–	–
>12 weeks	3	−2.99 (−6.53, 0.55)	0.530	0.00	0.435	–	–	–
Protein type						0.258		
Whey	6	**10.52 (2.07, 18.97)**	**0.015**	**96.6**	**<0.001**	–	–	–
Others	9	**5.17 (0.84, 9.50)**	**0.019**	**69.1**	**0.001**	–	–	–
Training status								
Untrained	8	6.12 (−0.74, 12.98)	0.081	**92.5**	**<0.001**	0.700		
Recreational	3	9.19 (−10.26, 28.64)	0.254	**84.1**	**0.002**	–		
Well-trained	4	**7.75 (4.44, 11.06)**	**<0.001**	15.8	0.313	–		
Protein time						**0.073**		
AE	7	4.64 (−4.36, 13.64)	0.312	**91.1**	**<0.001**	–	–	–
AE + BE	4	3.84 (−2.47, 10.14)	0.233	**82.1**	**0.001**	–	–	–
AE + BE+BS	2	**10.14 (6.42, 13.85)**	**<0.001**	0.00	0.404	–	–	–
Others	2	**20.55 (12.33, 13.85)**	**<0.001**	60.3	0.112	–	–	–

*p*^1^ value for effect; *p*^2^ value for heterogeneity between studies; *p*^3^ value for heterogeneity between groups with meta-regression; *p*^4^ value for publication bias (Begg’s test and Egger’s test, respectively). The bold values indicate statistically significant results (*p* < 0.05). CI, confidence interval.

**Figure 3:**
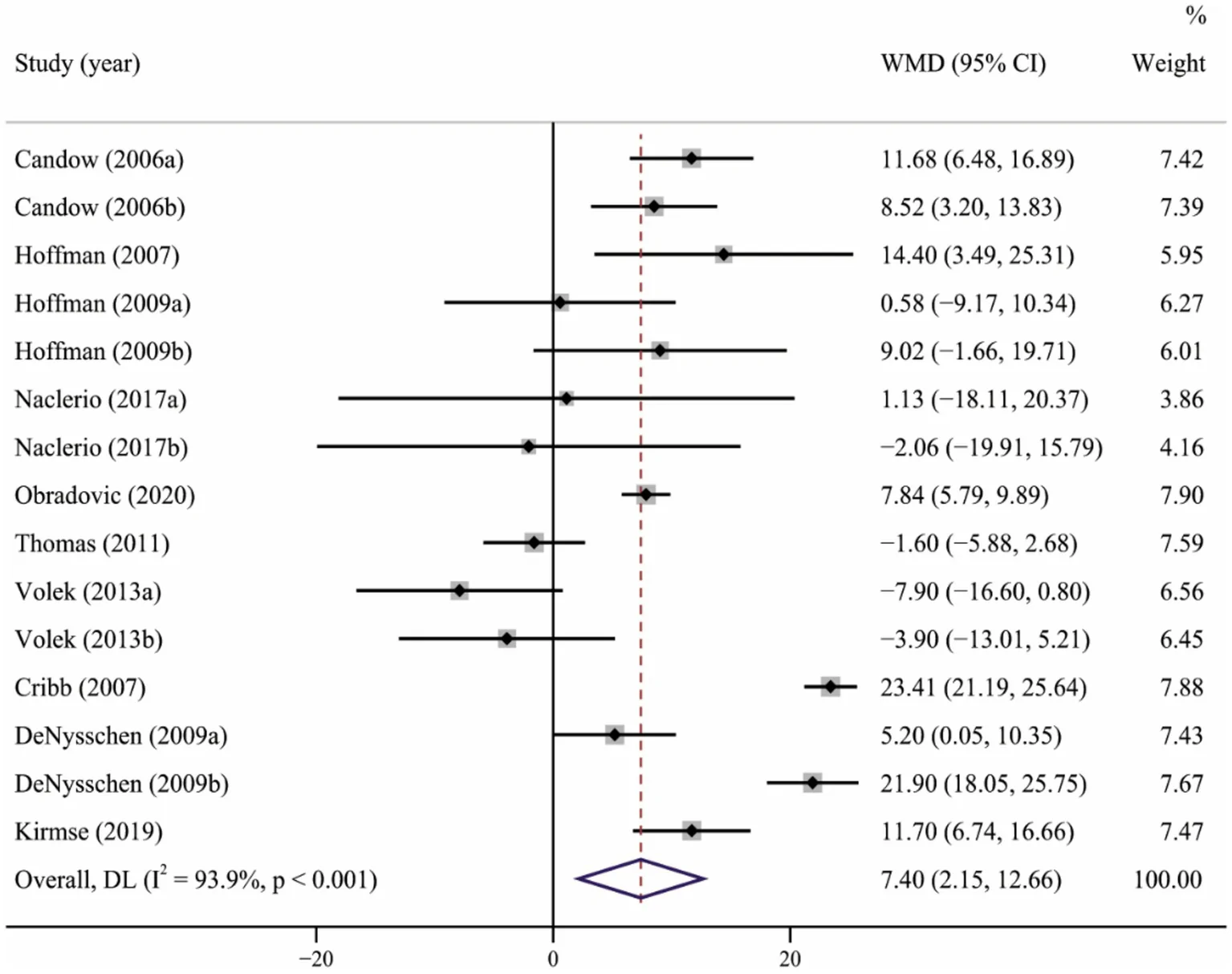
The forest plot of the effect of resistance training combined with protein supplementation on squat strength in young healthy adults.

### Subgroup analysis

The results of the subgroup analysis were presented in Table 2. The bench press results suggested that gender, duration, time of protein intake, and protein type could potentially contribute to significant heterogeneity in the study. Research conducted on both men and women revealed that the combination of protein intake and RT did not have a significant impact on the strength of bench press in young health adults, when compared to the control group. However, in studies involving individuals of mixed genders (WMD: 3.22 kg, 95% CI: 0.34–6.11, *p* = 0.028), the combination of protein intake and RT did lead to a significant improvement in bench press strength among young healthy adults. The combination of protein and RT intervention for a duration of ≤ 12 weeks (WMD: 2.89 kg, 95% CI: 0.81–4.96, *p* = 0.006) could significantly improve the bench press strength of young healthy adults, whey protein (WMD: 3.48 kg, 95% CI: 0.66–6.31, *p* = 0.015) and both around exercise (before and/or after exercise) and before sleep (AE + BE + BS) (WMD: 7.23 kg, 95% CI: 3.93–10.52, *p* < 0.001) could significantly improve the bench press of young healthy adults. From the results of squat strength, it could be seen that compared with the control group, protein intake combined with RT could significantly improve squat strength in young healthy adults in men studies (WMD: 10.64 kg, 95% CI: 4.47–16.81, *p* = 0.001) and studies with intervention periods of ≤12 weeks (WMD: 10.64 kg, 95% CI: 5.42–15.86, *p* = 0.001), as well as studies in which protein was supplemented around AE + BE + BS (WMD: 10.14 kg, 95% CI: 6.42–13.85, *p* < 0.001). Moreover, different types of protein intake could significantly improve the squat strength of young healthy adults. Therefore, gender, duration, protein intake time, and protein type may be sources of high heterogeneity. When stratified by pre-intervention resistance-training status, protein supplementation combined with RT significantly improved bench press strength in untrained participants (WMD: 1.92 kg, 95% CI: 0.46–3.38, *p* = 0.010), but not in recreational and well-trained participants. For squat strength, the effect was significant in well-trained participants (WMD: 7.75 kg, 95% CI: 4.44–11.06, *p* < 0.001), but not in untrained and recreational participants.

### Meta-regression analysis

To further identify potential sources of heterogeneity, meta-regression analysis showed that intervention duration was a statistically significant moderator of the effect of protein combined with resistance exercise on squat strength in healthy adults (*p* = 0.037), whereas protein timing showed a borderline non-significant trend (*p* = 0.073) (Table 2).

### Publication bias

As shown in Table 2, the results of Begg’s test, Egger’s test, and funnel plot analysis indicated that there was no publication bias in the results of the bench press strength (Figure 4, *p*_B_ = 0.697, *p*_E_ = 0.055) and squat strength (Figure 5, *p*_B_ = 0.882, *p*_E_ = 0.125).

**Figure 4:**
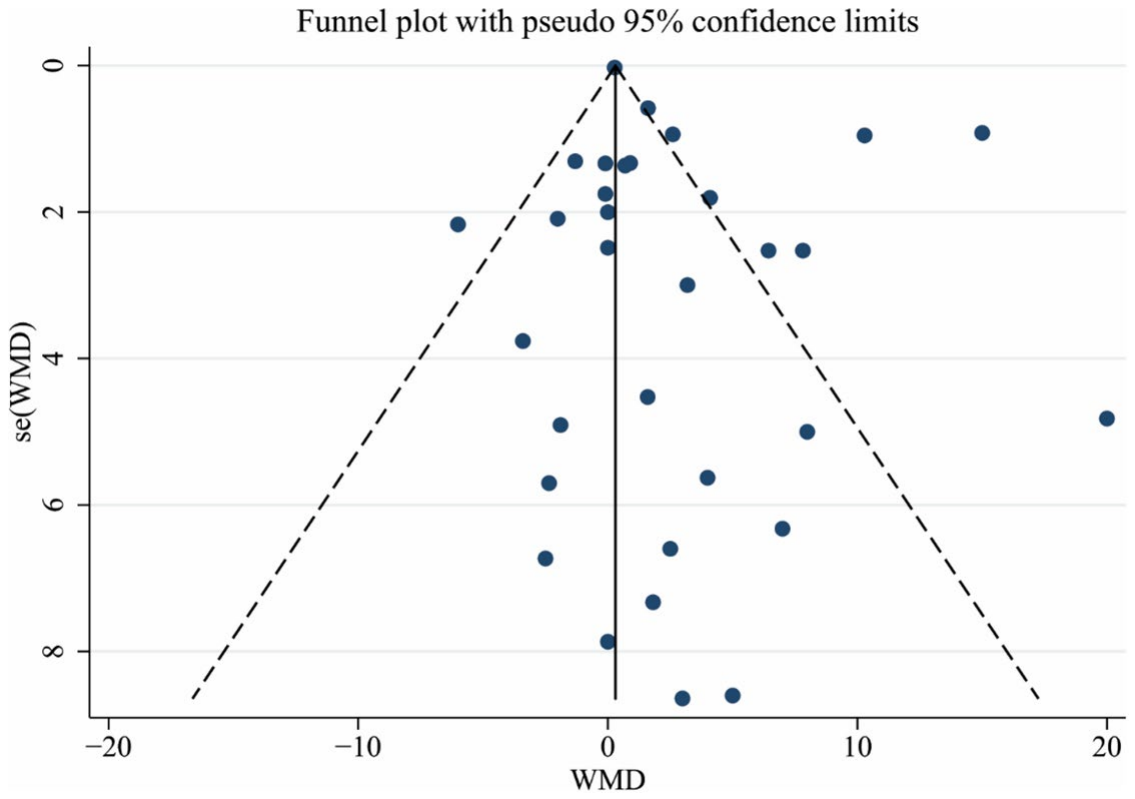
The funnel plot of resistance training combined with protein supplementation for bench press strength in young healthy adults.

**Figure 5:**
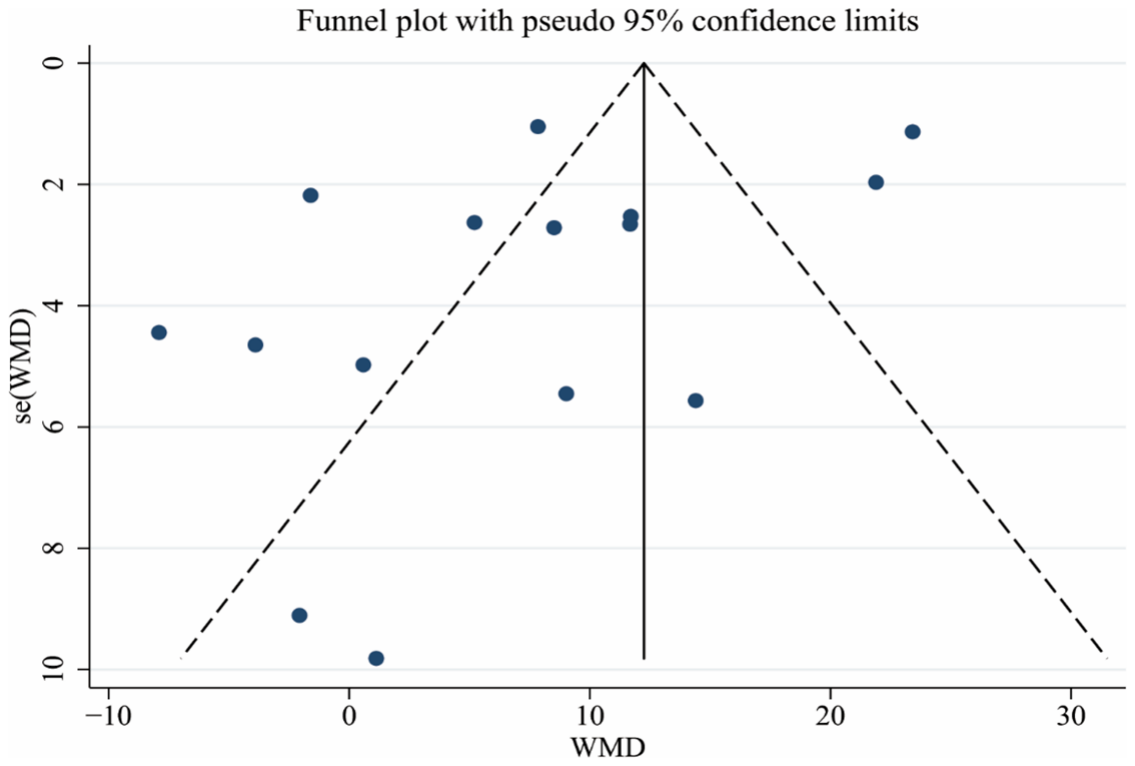
The funnel plot of resistance training combined with protein supplementation for squat strength in young healthy adults.

### Sensitivity analysis

Sensitivity analyses were conducted to evaluate the robustness of the pooled effects. First, in the leave-one-out meta-analysis, exclusion of any individual study did not materially alter the direction, magnitude, or statistical significance of the pooled effects for either bench press or squat strength (Supplementary Figures S1, S2). Second, to address the potential confounding effect of heterogeneous protein sources, leave-one-category-out sensitivity analyses were performed by excluding (1) studies using non-dairy or non-whey protein sources and (2) studies using mixed-protein formulations. In both analyses, the pooled effects remained statistically significant for bench press and squat strength, with effect directions consistent with the primary analysis (Table 4). Third, to assess the potential dependency arising from shared control groups, a sensitivity analysis was performed by dividing the shared control group sample size equally across comparisons for multi-arm studies. After this adjustment, the pooled effects remained statistically significant for both bench press (WMD: 2.76 kg, 95% CI: 0.77–4.75, *p* = 0.007) (Supplementary Figure S3) and squat strength (WMD: 7.73 kg, 95% CI: 2.31–13.15, *p* = 0.005) (Supplementary Figure S4), with heterogeneity similar to the primary analysis. Meta-regression findings were also consistent: for bench press, neither duration (*p* = 0.830) nor timing (*p* = 0.626) was significant; for squat strength, duration remained significant (*p* = 0.040) and timing remained borderline (*p* = 0.088) (Supplementary Table S2). Taken together, these findings indicate that the pooled effects were robust to the exclusion of non-dairy/non-whey and mixed-protein studies and to the adjustment for shared control groups, supporting the reliability of the main conclusion. However, heterogeneity remained high across all analyses, and the pooled estimates should therefore be interpreted with caution.

### Evidence level rated by GRADE

According to the GRADE guideline, it is displayed that protein supplementation significantly improves both bench press (moderate evidence) and squat strength (high evidence). While bench press results showed meaningful effects (+1) despite some inconsistency (−1), squat strength demonstrated significant improvements (+1 effect size) with explainable heterogeneity (+1). These findings confirm protein’s effectiveness for RT, particularly for lower-body exercises where intervention factors were clearly identified. (Table 3).

**Table 3:** Grades of Recommendation, Assessment, Development and Evaluation (GRADE) quality of evidence.

Outcome	Risk of bias	Inconsistency	Indirectness	Imprecision	Publication bias	Effect size	Plausible residual confounding	Dose–response gradient	GRADE rating
Bench press (1RM)	0	−1^a^	0	0	0	+1^b^	0	0	Moderate
Squat (1RM)	0	−1^a^	0	0	0	+1^b^	+1^c^	0	High

^a^Significant and unexplained variability exists in the overall meta-analysis. ^b^Find a significant effects size. ^c^Find significant source of heterogeneity that can be explained in the meta-analysis.

**Table 4:** Leave-one-category-out sensitivity analysis excluding non-dairy/non-whey protein studies and mixed-protein studies.

Outcomes (1RM)	Interventions	Mean difference (95% CI)	*p^1^* (effect)	Heterogeneity	
				I^2^ (%)	*p* ^2^
Bench press					
Excluding non-dairy/non-whey	29	**3.28 (1.26, 5.30)**	**0.001**	**92.8**	**<0.001**
Excluding mixed-protein studies	21	**3.63 (0.26, 6.99)**	**0.035**	**92.1**	**<0.001**
Squat					
Excluding non-dairy/non-whey	14	**7.01 (1.36, 12.67)**	**0.015**	**94.4**	**<0.001**
Excluding mixed-protein studies	10	**8.13 (1.89, 14.37)**	**0.011**	**95.9**	**<0.001**

*p*^1^ value for effect; *p*^2^ value for heterogeneity between studies; significant *p*-values (*p* < 0.05) are highlighted in bold prints. CI, confidence interval.

## Discussion

The present meta-analysis of 23 RCTs provides evidence that protein supplementation combined with RT significantly enhances muscle strength in young, healthy adults, although heterogeneity was high across studies. Overall, we found that protein supplementation combined with RT intervention can significantly improve both upper-body (bench press) and lower-body (squat) strength in resistance-trained young healthy adults.

The pooled estimates observed in the present meta-analysis—2.63 kg for bench press and 7.40 kg for squat—should be interpreted in relation to the typical strength levels and training status of the populations studied, as well as the measurement error of 1RM testing. In untrained young adults, baseline 1RM bench press values commonly range from approximately 40–70 kg depending on sex and body mass; thus, a 2.63 kg improvement corresponds to roughly 4–7% of baseline performance. Although this magnitude is not large, it exceeds the typical test–retest variability of 1RM testing (coefficient of variation approximately 1.86–9.32%) (46), suggesting that the effect is unlikely to be attributable to measurement error alone. For healthy trainees aiming at athletic performance or daily function, a 4–7% improvement in bench press may translate into a practically meaningful increase in training load (e.g., progressing from 60 kg to 62.5–65 kg), which is operationally relevant within a progressive overload framework. For squat, baseline 1RM values in untrained young adults typically range from approximately 60–100 kg, so a 7.40 kg improvement corresponds to roughly 7–12% of baseline performance. This exceeds the minimal clinically important difference (MCID) thresholds reported for clinical populations following pulmonary rehabilitation (approximately 5.7 kg for isotonic quadriceps muscle strength) (47). Moreover, ACSM guidelines recommend increasing resistance by 2–10% when the current load can be performed for 1–2 repetitions beyond the target (48). For a lifter with a baseline squat 1RM of 80–100 kg, this corresponds to an increment of approximately 2–10 kg per progression step, suggesting that the observed 7.40 kg improvement represents a meaningful training-load progression.

Additionally, all trials included in this meta-analysis employed a design in which protein was supplemented on top of the habitual diet, yet the majority did not report or standardize participants’ habitual dietary protein intake at baseline. This gap has important implications for the practical interpretation of our findings. The incremental benefit of protein supplementation is not universally applicable but depends heavily on an individual’s pre-existing total daily protein intake. Morton et al. showed that once total daily protein intake exceeds approximately 1.62 g/kg/day, additional protein supplementation no longer further promotes resistance training-induced gains in lean body mass (17). From a mechanistic perspective, when baseline dietary protein intake already approaches or reaches the saturation threshold for muscle protein synthesis (MPS), the additional anabolic stimulus provided by extra protein is likely to be limited; conversely, when baseline intake is insufficient, supplementation is more likely to produce observable strength gains (49, 50). Future studies should prioritize repeated measures of habitual dietary protein intake (e.g., 3–7 day dietary records) and employ stratified designs based on baseline intake levels (e.g., below, near, or above 1.6 g/kg/day) to clarify the interaction between baseline protein intake and protein supplementation.

Meta-regression analysis revealed that intervention duration was a statistically significant moderator of squat strength heterogeneity, while protein timing showed a borderline moderating trend. Subgroup analysis further suggested that the augmenting effect of protein supplementation on squat strength was significant when the intervention duration was ≤12 weeks, or when protein was supplemented both around exercise (pre- and/or post-exercise) and before sleep. However, because the meta-regression result for protein timing did not reach conventional statistical significance and some timing subgroups included a limited number of studies, these timing-related findings should be interpreted cautiously and considered hypothesis-generating.

Our study aligned with prior research findings indicating that the combination of protein supplementation and RT could significantly improve muscle strength in healthy adult subjects (51). There was evidence indicating that MPS (muscle protein synthesis) was a key factor that strongly influences the breakdown of muscle protein in healthy persons. Additionally, the combination of protein supplementation and RT could substantially enhance the rate of MPS (52). Compared to the RT intervention alone or placebo control group, the MPS levels were stimulated by protein supplementation combined with RT intervention by 40–150% higher than rest levels (53, 54). In a study of the dose–response relationship between protein intake and protein synthesis rate, it was found that the MPS rate increased as the protein intake increased (55). In addition, a study demonstrated that the mechanism by which protein supplementation combined with RT intervention enhanced muscle strength might be as follows: it maximizes the availability of amino acids, inhibits muscle protein breakdown caused by exercise, enhances the remodeling and repair of existing muscle proteins, as well as new MPS, and improves the adaptability of skeletal muscles to protein intervention combined with RT stimulation response, thereby enhancing individual muscle strength (56).

A key contribution of this study is the identification of intervention duration and protein timing as key moderators contributing to heterogeneity in squat strength outcomes. Consistent with the existing evidences, 12 weeks or less of protein supplementation combined with RT can significantly increase muscle strength in young healthy adults. Notably, this ergogenic effect was attenuated in studies extending beyond 12 weeks, suggesting that prolonged interventions may not yield additional strength benefits. This observation aligns with the established physiological principle of diminishing returns in resistance-trained individuals, wherein neuromuscular adaptations reach a plateau following extended training periods (57). Consequently, the relative contribution of protein supplementation becomes less pronounced as training duration increases (58–60). This phenomenon could explain why studies exceeding 12 weeks failed to show significant additive effects compared to RT alone.

The critical role of supplementation timing—particularly peri-exercise and pre-sleep intake—offers practical insights. Pre−/post-exercise protein likely maximizes acute anabolic opportunities by elevating muscle protein synthesis (MPS) during the “anabolic window,” while protein intake before sleep may counteract catabolic processes during prolonged fasting (61). The findings of our research indicated that the consumption of protein prior to bedtime may significantly improve muscle strength in young healthy adults. This phenomenon may be attributed to the following factors: protein intake during sleep produced the same dynamics of protein digestion and absorption as during the day, indicating that intestinal function remains normal at night. Protein intake might be used as a nutritional strategy to promote plasma amino acid availability and MPS during nighttime sleep, thereby promoting the positive balance of nighttime muscle protein (62). There is evidence to suggest that individuals who consume a substantial quantity of protein (20-30 g) at night have a higher lean leg mass and knee extensor strength than those who consume protein in the afternoon (63). Furthermore, protein intake before bed (casein) combined with RT resulted in a 37 percent higher myofibrillar myoglobin synthesis compared to bedtime protein intake alone (64). Several studies have demonstrated that the protein intake group had higher systemic MPS rates during sleep convalescence than the control group when comparing the difference in convalescent MPS after nocturnal protein intake or placebo supplementation combined with RT (65, 66). Several long-term studies have also examined the impact of protein consumption prior to sleep on muscle mass. The findings demonstrated that consuming protein before going to sleep could have a substantial impact on increasing the size of the quadriceps muscles, the area of type II muscle fibers, and muscle strength (67) Additionally, it could also lead to improvements in total body composition and the amount of lean body mass in the legs (68). It can be seen that during the relatively long nutritional gap period at night, protein intake before bed may serve as a new nutritional supplement window to promote MPS more effectively and maintain protein balance at night. Nonetheless, given the borderline meta-regression result and the limited number of studies in the AE + BE + BS subgroup, these timing-related conclusions should be interpreted with caution and need confirmation by future well-powered RCTs.

Several limitations should be acknowledged. First, heterogeneity was high for both bench press and squat strength, and although intervention duration was a significant moderator and protein timing a borderline moderator for squat strength, residual unexplained variability remained. Second, a dose–response meta-regression using daily supplemental protein dose was not feasible because the included trials did not provide low-, moderate-, and high-dose comparison groups. Third, some subgroup analyses, particularly for protein timing and training status, included few interventions, and the borderline result for protein timing limits the strength of timing-related conclusions. Fourth, most trials involved male participants and did not report or standardize baseline dietary protein intake, limiting generalizability to females and interpretation against habitual protein status. Finally, some studies contributed multiple intervention arms sharing the same control group; although control participants were not double-counted in the pooled sample size, the same control data were used in multiple comparisons, which may overestimate the precision of the pooled estimates.

## Conclusion

In conclusion, protein supplementation combined with resistance training significantly improved bench press and squat strength in young healthy adults. Future well-powered randomized controlled trials with standardized protein dose and source reporting, repeated measures of habitual protein intake, and stratified designs are needed to clarify the interaction effects of training status, supplementation timing, and baseline protein status.

## Data Availability

The original contributions presented in the study are included in the article/Supplementary material, further inquiries can be directed to the corresponding authors.
