# Supplementary material for: Protein supplementation and resistance training-induced strength gains in young adults: a meta-analysis
Source: Front Nutr. 2026 Oct 13;13:1946052. doi: 10.3389/fnut.2026.1946052 (PMC13710401; doi:10.3389/fnut.2026.1946052)
Supplement: Supplementary file 2 [file Table_2.DOCX]

**Supplementary table 1 Detailed search strategy**

Search strategy for the PubMed database.

**((Dietary Proteins[MeSH]) OR (Proteins, Dietary[Title/Abstract]) OR (proteins[MeSH]) OR (diet[MeSH]) OR (Dietary Supplements[MeSH]) OR (Dietary Supplementations[Title/Abstract]) OR (Food Supplements[Title/Abstract]) OR (Food Supplementations[Title/Abstract]) OR (Nutraceuticals[Title/Abstract]) OR (supplement[Title/Abstract]) OR (protein consume[Title/Abstract]) OR (protein intake[Title/Abstract])** OR (whey**[Title/Abstract]**) OR (casein**[Title/Abstract]) OR (soy[Title/Abstract]) AND ((Resistance Training[MeSH]) OR (Resistance exercise[Title/Abstract]) OR (Weight Lifting[Title/Abstract]) OR (Strengthening Program[Title/Abstract]) OR (Strength Training[Title/Abstract]) OR (Weight-Bearing[Title/Abstract]) OR (Exercise Program[Title/Abstract]) OR (Isometric Contraction[MeSH]) OR (**Squat **[Title/Abstract]) OR (**Bench press **[Title/Abstract]) OR (**muscle strength **[Title/Abstract]) OR (Muscle function[Title/Abstract])) AND ((randomized controlled trial [Publication Type]) OR (randomized) OR (placebo))**

Search strategy for Web of Science and SPORTDiscus databases

**(“Dietary Proteins” OR “Proteins, Dietary” OR “proteins” OR “diet” OR “Dietary Supplements” OR “Dietary Supplementations” OR “Food Supplements” OR “Food Supplementations” OR “Nutraceuticals” OR “supplement” OR “protein intake” OR “intake” OR “protein consume”** OR “whey**”** OR “casein**” OR “soy”) AND (“Resistance Training” OR “Resistance Exercise” OR “Weight Lifting” OR “Strengthening Program” OR “Strength Training” OR “Weight-Bearing” OR “Exercise Program” OR “Isometric Contraction” OR “**Bench press**” OR “**Squat**” OR “**muscle strength**” OR “Muscle function”) AND (“randomized controlled trial” OR “randomized” OR “placebo”)**

Search strategy for Cochrane Library databases.

**(“Dietary Proteins” OR “Proteins, Dietary” OR “proteins” OR “Dietary Supplements” OR “Dietary Supplementations” OR “Food Supplements” OR “Food Supplementations” OR “Nutraceuticals” OR “protein intake” OR “protein consume”** OR “whey**”** OR “casein**” OR “soy”) AND (“Resistance Training” OR “Resistance Exercise” OR “Weight Lifting” OR “Strengthening Program” OR “Strength Training” OR “Weight-Bearing” OR “Exercise Program” OR “Isometric Contraction” OR “**Bench press**” OR “**Squat**” OR “**muscle strength**” OR “muscle function”) AND (“randomized controlled trial” OR “randomized” OR “placebo”) in Title Abstract Keyword - in Trials (Word variations have been searched**

**Supplementary Figure 1** Sensitivity analysis results of resistance training combined with protein supplementation on bench press strength in young healthy adults.

**Supplementary Figure 2** Sensitivity analysis results of resistance training combined with protein supplementation on squat strength strength in young healthy adults.

**Supplementary Figure 3** Sensitivity analysis forest plot of the effect of resistance training combined with protein supplementation on bench press after dividing the shared control group sample size equally across comparisons.

**Supplementary Figure 4** Sensitivity analysis forest plot of the effect of resistance training combined with protein supplementation on squat strength after dividing the shared control group sample size equally across comparisons.

**Supplementary table 2** Meta-regression results stratified by study characteristics after equally dividing the shared control group sample size across comparisons

| **Outcomes (1RM)** | **Interventions** | **Mean difference (95% CI)** | ***P^1^* (effect)** | **Heterogeneity** | | ***P*^3^(meta-regression)** |
| --- | --- | --- | --- | --- | --- | --- |
|  |  |  |  | **I^2^ (%)** | ***P*^2^** |  |
| **Bench press** |  |  |  |  |  |  |
| **Overall** | 32 | **2.76 (0.77, 4.75)** | **0.007** | **92.7** | **< 0.001** | **-** |
| **Duration** |  |  |  |  |  | 0.830 |
| **Protein time** |  |  |  |  |  | 0.626 |
| **Squat** |  |  |  |  |  |  |
| **Overall** | 15 | **7.73 (2.31, 13.15)** | **0.005** | **93.3** | **< 0.001** | - |
| **Duration** |  |  |  |  |  | **0.040** |
| **Protein time** |  |  |  |  |  | **0.088** |

*P^1^*value for effect; *P^2^* value for heterogeneity between studies; *P^3^* value for heterogeneity between groups with meta-regression; Abbreviations: CI, conﬁdence interval.
